# Nanomechanics of individual aerographite tetrapods

**DOI:** 10.1038/ncomms14982

**Published:** 2017-04-12

**Authors:** Raimonds Meija, Stefano Signetti, Arnim Schuchardt, Kerstin Meurisch, Daria Smazna, Matthias Mecklenburg, Karl Schulte, Donats Erts, Oleg Lupan, Bodo Fiedler, Yogendra Kumar Mishra, Rainer Adelung, Nicola M. Pugno

**Affiliations:** 1 Institute of Chemical Physics, University of Latvia, Raina bulvāris 19, LV-1586 Rīga, Latvia; 2 Laboratory of Bio-Inspired & Graphene Nanomechanics, Department of Civil, Environmental and Mechanical Engineering, University of Trento, via Mesiano 77, I-38123 Trento, Italy; 3 Functional Nanomaterials, Institute for Materials Science, Kiel University, Kaiserstraße 2, D-24143 Kiel, Germany; 4 Institute for Polymers and Composites, Hamburg University of Technology, Denickestraße 15, D-21073 Hamburg, Germany; 5 School of Engineering and Materials Science, Queen Mary University of London, Mile End Road, London E1 4NS, UK; 6 Ket-Lab, Italian Space Agency, via del Politecnico snc, I-00133 Roma, Italy

## Abstract

Carbon-based three-dimensional aerographite networks, built from interconnected hollow tubular tetrapods of multilayer graphene, are ultra-lightweight materials recently discovered and ideal for advanced multifunctional applications. In order to predict the bulk mechanical behaviour of networks it is very important to understand the mechanics of their individual building blocks. Here we characterize the mechanical response of single aerographite tetrapods via *in situ* scanning electron and atomic force microscopy measurements. To understand the acquired results, which show that the overall behaviour of the tetrapod is governed by the buckling of the central joint, a mechanical nonlinear model was developed, introducing the concept of the buckling hinge. Finite element method simulations elucidate the governing buckling phenomena. The results are then generalized for tetrapods of different size-scales and shapes. These basic findings will permit better understanding of the mechanical response of the related networks and the design of similar aerogels based on graphene and other two-dimensional materials.

## Abstract (web summary)

Aerographite is a highly porous and lightweight carbon material obtained from hollow tubular tetrapod building units. Here, the authors present a comprehensive investigation of tetrapod deformation mechanisms which are at the core of aerographite nanomechanical properties.

Three-dimensional (3D) cellular materials built from carbon nanostructures are currently under increasing investigation in terms of fabrication and physical properties because of their significant technological potential for diverse advanced applications, such as biological scaffolds, electrochemical biosensing, supercapacitors, light weight flexible batteries, and highly efficient oil absorbers^1^,^2^,^3^,^4^,^5^,^6^,^7^,^8^,^9^. Since the introduction of graphene, a large variety of synthesis methods, involving direct growth, wet chemistry and templates, have been employed for the growth of 3D carbon-based nanomaterials^9^,^10^,^11^,^12^,^13^,^14^,^15^ and the field is still under development. Nanoscale carbon structures can be built, for example, from carbon nanotubes (CNTs) or graphene flakes^16^ exhibiting very high surface to volume ratios from which interesting physical and chemical features originate. But to efficiently access their unique nanoscopic features, these materials should be preferentially available in a macroscopic 3D form with a sufficient mechanical robustness and stability so that they can be manufactured into any desired structured shape^17^. Thus, 3D carbon-based networks comprising both sufficient mechanical strength and very high porosity are desirable, but this is a challenging task in 3D cellular networks. For instance, it is well known that 3D networks based on CNTs being randomly interconnected and held in place only by van der Waals (vdW) forces are prone to failure when compressed and several attempts have been made to overcome this drawback^18^,^19^,^20^. In this context, the morphology and interconnections of the nanoscale carbon-based network building units play a fundamental role.

As basic building block of porous 3D networks, the tetrapod geometry is an interesting shape, since when accumulated together their spatially extended arms can prohibit close packing very efficiently. Recently, a new concept for producing tetrapod-based 3D networks has been introduced by the flame transport synthesis of zinc oxide^21^,^22^,^23^. During the re-heating at high temperatures the nano- and micro-scale tetrapod arms build interconnections, forming a bridging 3D network which provides necessary mechanical strength and simultaneously very high porosity (up to ∼98% just by controlling the initial tetrapod template amount in the scaffold)^21^,^24^. Furthermore, if these ZnO tetrapods are loaded with other metal oxide nanostructures, hybrid 3D interconnected networks can be easily realized^25^, which are suitable for different applications^26^. In the context of carbon-based networks, the ZnO can also be exploited as sacrificial template for the growth of ultra-lightweight and highly porous (porosity>99.99%) 3D multilayer aerographite (AG) networks (also known in the literature as aerographene). In a single-step chemical vapour deposition (CVD) process it is possible to form hollow nano- and micro-tubular multilayer graphene structures, which adopt basic features of the shape of the ZnO templates. The high temperature, together with the presence of a carbon source and of hydrogen (carbon by precursor and hydrogen by gas) allow the deposition of nm-thick graphene flakes on ZnO (ref. 27). Then ZnO is chemically reduced to metallic Zn, whereas Zn evaporates and is removed by the carrier gas (Ar). This process had been introduced in 2012 together with model of a belt-like growth^27^. Meanwhile, the working principle of the CVD synthesis was confirmed by other authors^28^,^29^,^30^. By modifying the CVD parameters several variants can be synthesized, which differ in walls morphology (closed or open) and/or inner graphitic fillings^27^. Some further AG variants do not have closed graphene-based shells, but just consist of narrow carbon filaments on the former tetrapod surfaces and thus possess an extreme high level (>99.99%) of porosity^27^. This kind of hierarchical networks containing carbon filaments is even more attractive in terms of porosity and was used to realize flexible and semiconducting composites which could be exploited as next generation materials for electronic, photonic and sensors applications^17^,^31^.

Apart from being highly porous and extremely lightweight, AG exhibits very interesting specific mechanical properties such as remarkable specific tensile strength (σ/*ρ*) and Young's moduli (*E*/*ρ*)^27^, being in principle ideal candidates for impact protection and shock absorption. Some variants show also self-stiffening in cyclic mechanical loading^27^. To use AG for different applications, and thus predict the overall mechanical properties of its 3D networks, a detailed understanding of the mechanical behaviour of its individual building blocks is necessary. So far, even nanoscale hollow carbon tetrapods synthesized using ZnO tetrapods templates have been studied with respect to their different properties but their constitutive mechanical response has not been discussed yet.

Here we investigate and model the mechanical behaviour of single tetrapods, building a base for the future prediction of the mechanical behaviour and properties of AG networks with different densities and known tetrapod characteristics. Single arms of AG tetrapods were bent *in situ* (with other arms fixed) inside a scanning electron microscope (SEM) in a controlled manner by a soft atomic force microscopy (AFM) cantilever. Buckling of the central joint was seen to be the governing mechanism of the behaviour of the single tetrapod. We then propose an analytical nonlinear nanomechanical model for describing the formation of a `̀buckling hinge'' at the tetrapod central joint or within the arms. Complementary, finite element method (FEM) simulations were carried out, assuming identical conditions of the tetrapod arms corresponding to the *in situ* atomic force and scanning electron microscopy experiments. The results from *in situ* experiments, analytical calculations and FEM simulations are in good agreement. The observed internal tetrapod deformation mechanisms and the constitutive behaviour are then generalized for different size-scales and shapes, that is tube cross-section aspect ratios. Thus, the results can help in the design of aerogel tetrapodal networks of different materials and structures.

## Results

### Morphology of aerographite tetrapods

**Figure 1:**
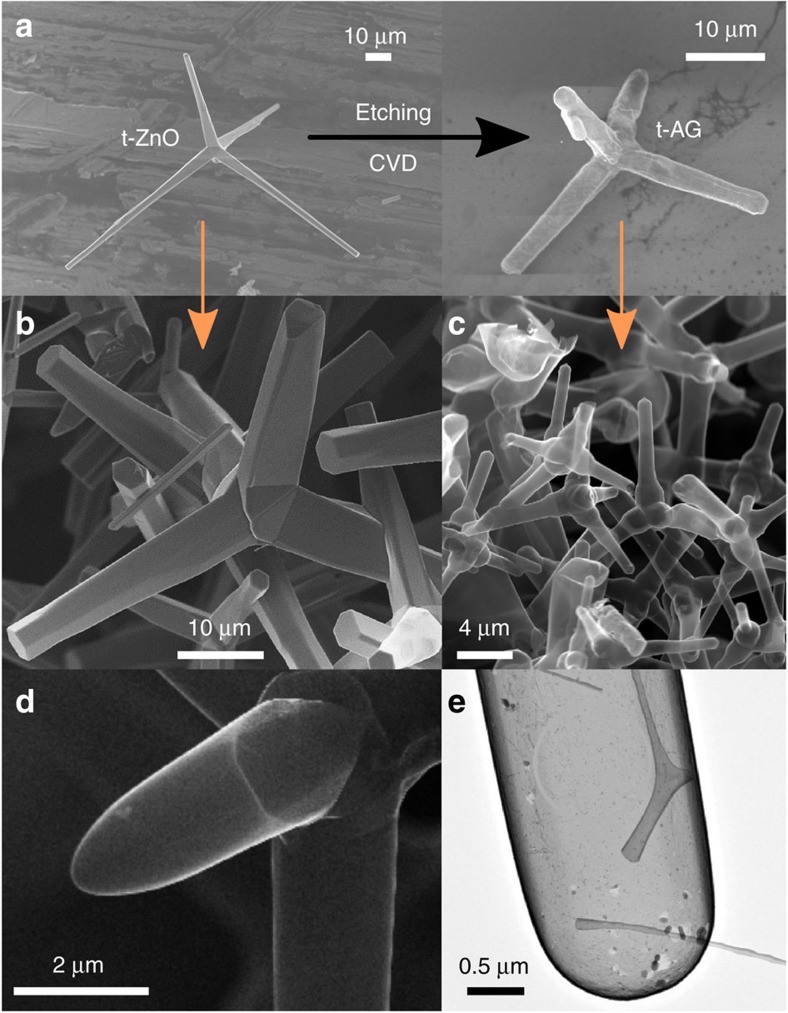
Production of AG tetrapods. (**a**) Schematic illustration of the formation of t-AG from sacrificial tetrapodal ZnO (t-ZnO) in the CVD process. (**b**,**c**) Typical high-resolution SEM images corresponding to t-ZnO (left) and converted t-AG networks (right), respectively. (**d**) Further high-resolution SEM image from the tip and middle of a t-AG arm. (**e**) TEM bright field image of an AG tube with closed walls.

First, the morphology of the AG tetrapods was studied. In Fig. 1a the conversion principle of tetrapodal ZnO into hollow and tubular tetrapodal AG (t-AG) during the CVD process is schematically illustrated. Furthermore, the panel displays representative SEM images of a typical ZnO tetrapod (Fig. 1b) and of an AG tetrapod (Fig. 1c), respectively, before and after the CVD conversion within the network. The ZnO tetrapod template used in this study, and thus the resulting graphene counterpart, has four arms which are interconnected together with a mutual dihedral angle of ∼106° via a central joint, resulting in a 3D spatial shape^27^. Thus, their geometry can be defined, in a good approximation, from the vertexes and the centroid of a regular tetrahedron. In the variant used here, the ZnO tetrapods exhibit uniform hexagonal cylindrical arms narrowing towards their tips (see SEM image in Fig. 1a). The arms typically have a diameter of ∼1, 5 μm at their tip and joint, respectively. The arm length is in the range of 15–30 μm. The morphology of the t-AG arms is strongly influenced by the growth parameters during the CVD process and, if required, t-AG variants with hollow tubular arms, a closed shell and low aspect ratios can be grown^27^. The arms of the AG tetrapods used for the *in situ* bending experiments exhibit a hollow tubular morphology with diameters being equally in the range of 1–5 μm and wall overall thicknesses of the graphene flakes of ∼15 nm, see energy filtered transmission electron microscopy (TEM) picture in the Supplementary Fig. 1. The AG variant which was used here, possesses tubular and smooth walls (see t-AG SEM images in Fig. 1c–e). Other crumpled variants may arise from thickness-dependent growth processes which could be influenced by several parameters, such as surface energies, defects and internal mechanical stresses during the conversion process in the CVD chamber.

### Tetrapod bending experiments

**Figure 2:**
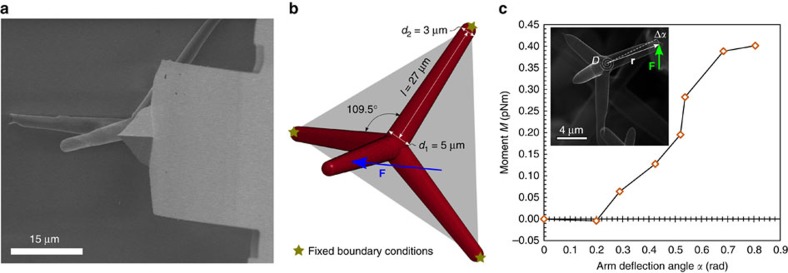
Bending experiment on individual tetrapod attached to silica substrate. (**a**) SEM image of the tested tetrapod under bending action of an AFM cantilever BL-RC-150VB from Olympus (spring constant *k*=2.9–50 pN nm^−1^). As the cantilever is moved from right to left parallel to the substrate, both the arm of the tetrapod and the cantilever are bent (see Supplementary Movie 1). (**b**) FEM model with detail of the geometry of the tetrapod reported in **a** (the tetrapod is assumed with extreme points corresponding to the vertexes of a regular tetrahedron). (**c**) From the AFM acquired raw data (applied force and cantilever deflection as schematically depicted in the inset picture) the current applied moment *M* and corresponding arm rotation angle *α* are determined (see Supplementary Note 1).

During the first type of *in situ* experiment a tetrapod arm was bent inside a SEM with a soft AFM cantilever while the other three arms were attached to a substrate, following a well-established procedure^32^,^33^,^34^. It was repeatedly observed via instant video recording (Supplementary Movie 2), that the free standing AG tetrapod arms tend to preferentially rotate around the central joints and to experience there localized elastic instability, while the arm itself behaves very rigidly. Thus the tetrapod joint appears to be the most compliant location of the arm, as demonstrated later. In general, when a tube starts to buckle its stiffness is significantly lowered^35^. To examine and quantify this stiffness reducing effect of the buckling-hinge, the free-standing AG tetrapod arm depicted in Fig. 2a,b was deflected to an angle *α*=0.6 rad with the help of the aforementioned AFM cantilever tip from the right towards left side of the image, parallel to the surface. The angle increment Δα at the buckling joint is a function of the resulting moment and of the joint rotational stiffness *D*, thus 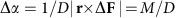 where **F** is the external applied force on one of the arms and **r** its lever arm with respect to the computing point (inset in Fig. 2c). Being in the elastic regime, we assume *D* as constant until the applied moment *M*=|**r**||Δ**F**|sin*θ* is lower than the buckling threshold *M*_bh_ which is a function of the joint cross-section geometry and of the material elastic properties. The resulting non-linear moment–rotation curve experimentally measured is shown in Fig. 2c. As expected, it reveals progressively decreasing rotational arm stiffness for higher deflection angles.

We propose a nonlinear equation for describing the formation of a buckling hinge in the tetrapod central joint or along the arm length analogously to the moment-rotation curve observed during the formation of a plastic hinge in elastic-plastic bent beams. In both cases (buckling and yielding) the involved sections at large load possess very low rotational stiffness: a large local deformation arises with small increment of load and the local curvature *χ* goes to infinite (the radius of curvature *ρ*=1/*χ*→0). Considering a homogeneous linear elastic perfectly plastic isotropic material, an initial linear regime occurs. Then, if the section is sufficiently thick and the material ductile, localized plastic deformation starts with the formation of the so-called plastic hinge^36^. On the contrary, if the section is very thin, as in the case of tubular sections, the local elastic buckling may forego yielding or fracture. When *M>M*_bh_ the following non-linear buckling-hinge equation enters into play (see ‘Methods' section for its derivation):


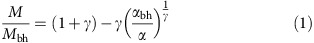


where *α*_bh_ is the joint (hinge) rotation at the buckling onset and 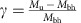 is the model parameter which describes the evolution of the hinge, with *M*_u_ being the maximum asymptotic bending moment that the hinge is able to carry. Note that the equation (1) is the generalization of the one describing the evolution of the bending moment after a formation of a plastic-hinge: for example when *γ*=1/2 it represents the plastic behaviour of a filled rectangular cross-section. The analogy holds just in the monotonic loading regime: in fact the buckling hinge can be completely reversible. Eventually, different values of *γ* could be estimated for different cross-section and different causes of the joint rotation.

**Figure 3:**
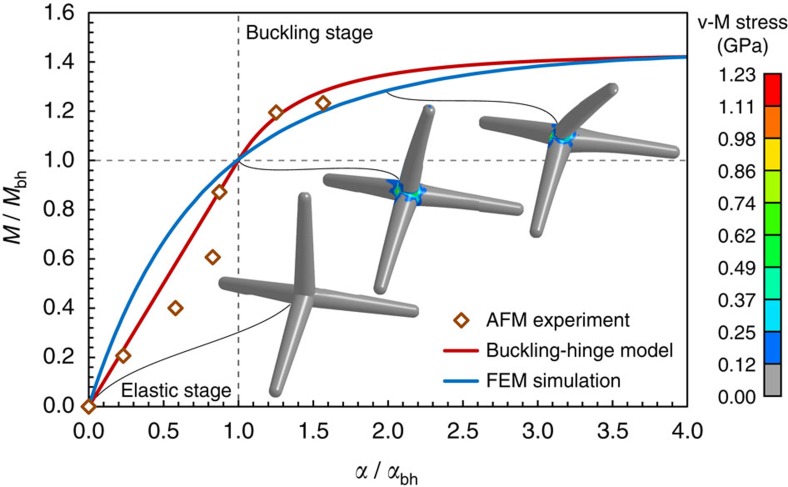
Normalized moment-rotation curve for bending of the tested single tetrapod. Experimental results (dots), buckling-hinge model fitted on experimental data (red line) and FEM simulation (blue line) are reported. Contour plots of the von-Mises stress in the tetrapod outer layer of the wall is plotted (scale bar in GPa) showing the stress concentration at the central joint.

We then simulated the *in situ* experiment presented in Fig. 2a in which the geometry of the tetrapod was highly regular and clearly visible from the SEM, being its bending not covered by the AFM cantilever. The length of each arm was derived from the *in situ* SEM videos (the detailed procedure is described in the Supplementary Note 1) and found to be ∼27 μm (Fig. 2b). This value was set as the distance from the base of the tetrapod arm (thus not the central joint) and the top face of the circular tapered cone defining the arm end (Fig. 2b). The diameters of the cone at the tetrapod central joint and at the arm end are respectively *d*_1_=5 μm and *d*_2_=3 μm and each tetrapod arm is capped at the end with a hemispherical shell of diameter *d*_2_ (Fig. 2b). We assumed a wall thickness *t*=15.3 nm, namely corresponding to 45 graphene layers, as suggested from an energy filtered TEM image taken from a representative tetrapod arm (see Supplementary Fig. 1). Figure 3 shows the normalized moment–rotation curves of the analysed tetrapod, comparing the experimental results with the curves defined by the non-linear buckling-hinge model and FEM simulation. The analytical curve is obtained from the best-fit of the experimental data (corresponding to the ones reported in Fig. 2), while the FEM simulations are calibrated assuming as fixed degrees of freedom, the buckling point (*M*_bh_, α_bh_) and the ultimate hinge moment *M*_u_. From the buckling-hinge model we estimated *γ*=0.44, and *D*=0.85 pN m rad^−1^ in the elastic regime (*α*<*α*_bh_), while from FEM simulation we determined as best-fit of the AFM experiment a Young's modulus of *E*=270 GPa (refs 37, 38), which was not known *a priori*. Notice that the *γ* for a thin circular elastic–plastic section undergoing yielding would be *γ*≈0.27. FEM images of the tetrapod deformation at three different stages are depicted as inset in Fig. 3 displaying stress distribution within the tetrapods (see also Supplementary Movie 3). These FEM pictures confirm that, prior to buckling, the response is governed by a transverse deformation of the adjacent arms nearby the joint and that in the end it merges in the central joint buckling (see FEM third stage image of Fig. 3b) and that bending deformation of the loaded arm has a negligible contribution. This can also be theoretically claimed approximating the arm as a bent cantilever of length *l* under a concentrated force at the free end: indeed, assuming by absurd that the arm tip displacement 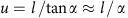 is due to the elastic bending of the arm, the materials Young's modulus can be derived as *E*=*lD*/(3*J*), where *J* is the average cross section moment of inertia of the tapered arm. The corresponding calculated value would be for our case *E≈*20 MPa, which is very low referring to nominal properties of multi-layer graphene^39^. Consequently, for our thin-walled tube tetrapods, the pure elastic arm bending is negligible when compared to the most compliant buckling-hinge section, either is represented by the central joint or by an intermediate arm section.

**Figure 4:**
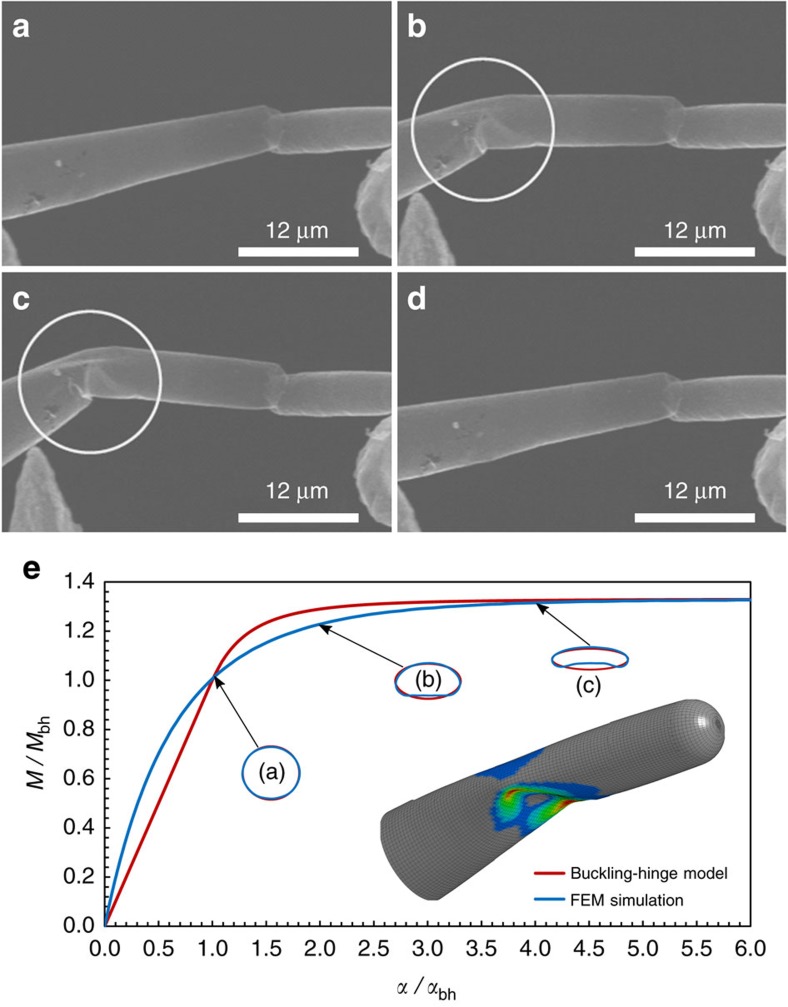
Reversible buckling of a bent AG tubular arm. (**a**) Tube in the undeformed state; (**b**) the tube has started to buckle (position indicated by the circle); (**c**) tube heavily buckled with its stiffness dramatically decreased; (**d**) the tube recovered elastically its original shape. (**e**) FEM simulation derived curve (blue) and the analytical one (red) determined from the buckling-hinge model are reported. The shape of the buckling-hinge cross section at different stages from simulation and its prediction from analytical calculations are depicted. The estimated buckling-hinge parameter is *γ*=0.33, note that the corresponding value determined for buckling at the tetrapod central joint was *γ*=0.44.

In a second type of *in situ* experiment the AG arm was isolated from the tetrapodal structure and thus from the central joint and was instead placed in between two gold tips (see Supplementary Fig. 2). In this way, without the AFM cantilever tip masking the buckling location, a better visual evaluation of the hinge formation was realized by constructing a deformation situation in which it was more probable to observe buckling at the most compliant position along the arm length. Figure 4 shows a series of SEM micrographs from the buckling of a single tube of AG bent in between the two gold tips, from the undeformed state (Fig. 4a) to a state in which the tube has started to buckle (position indicated by the circle in Fig. 4b), to a heavily buckled state in which the stiffness of the tube is dramatically decreased due to buckling (Fig. 4c). This confirms that buckling can occur even on the arm provided that the joint rotational stiffness is sufficiently high (here the joint is not present and the left-hand side extremity can be assumed fully clamped, thus analogous to a rigid joint). Interestingly, the tube recovered elastically to its original shape without any visible damage after flexure folding (Fig. 4d and Supplementary Movie 5). This property has already been reported by Falvo *et al*.^40^ for multiwalled CNTs, but in contrast to these CNTs the diameter of the examined AG tubes is about three orders of magnitude bigger. However, these results indicate similarities between the elastic buckling of AG tubes and multiwalled CNTs. The buckling-hinge model is likewise applicable to this case. We assumed that the buckled section takes an elliptic shape, this can be computed by imposing two conditions: (1) the perimeter of the tube section must keep constant under ovalization and (2) the cross-section moment of inertia is related step by step to the current value of the joint rotational stiffness *D*(*α*) (see ‘Methods' section for its analytical derivation). Figure 4e shows the curve obtained from FEM simulation and the fit obtained with the buckling-hinge model (*E*=270 GPa, *d*_1_=0.5 μm, *d*_2_=0.75 μm, *t*=15.3 nm and *l*=2.6 μm). The buckling-hinge model validity is also confirmed by the good agreement between the simulation observed cross-section shape at the buckling hinge and its analytically derived counterpart (Fig. 4e).

### Scaling laws

**Figure 5:**
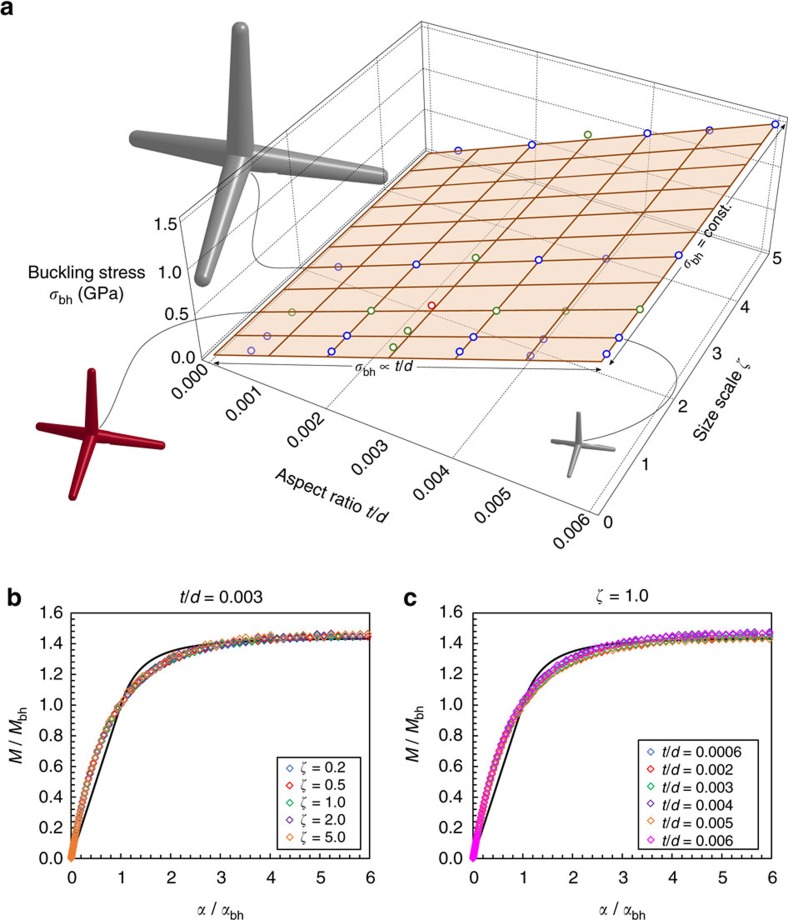
Scaling of the joint mechanical properties for different tetrapod size scales (*ζ*=*d*/*d*_0_=*l*/*l*_0_) and tube aspect ratios (*t*/*d*). (**a**) Maximum buckling stress *σ*_bh_=*E*ɛ_bh_ at the joint section from numerical simulations (dots) compared with the best-fit surface of equation (2). It emerges nearly independence of the buckling stress/strain from the size scale (*t*/*d*=const.) and linear dependence with respect to the aspect ratio *t*/*d.* The red dot represents the nominal tested tetrapod of Fig. 3 (*ζ*=1, *t*/*d*=0.003) while the green dots correspond to its size scaling with *t*/*d=*const.=0.003, or to the aspect ratio scaling only (*ζ*=1). Tetrapod at three different size scales (*ζ*=0.2, 1.0, 2.0) are depicted. (**b**) Dimensionless moment-rotation curves of the 5 performed simulations with *t*/*d=*const.=0.003 compared to the analytical prediction of the buckling-hinge model (continuous line). (**c**) Dimensionless moment-rotation curves of the 6 performed simulations with *ζ*=1 compared to the analytical prediction of the buckling-hinge model (continuous line).

These results can be generalized to tetrapods of different size-scale and shape, namely aspect ratio *t*/*d*. Indeed, it is acknowledged that for thin-walled tubes, such as ours, the critical compressive local strain *ɛ*_bh_, corresponding to the buckling condition under bending, is given by the following relation^41^,^42^:


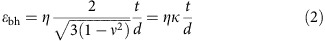


where, in our case, *κ*≈1.178 assuming a Poisson's ratio *ν*=0.2 for the graphite tube walls and *η* is an adimensional factor theoretically equal to 1. Figure 5a shows the simulations results in terms of critical buckling stress *σ*_bh_ for tetrapods of different scales *ζ*=*d*/*d*_0_=*l*/*l*_0_ both for constant and variable aspect ratios *t*/*d*. These are compared to the analytical predictions of equation (2), according to *σ*_bh_=*Eɛ*_bh_, from where a very good agreement is observed, considering *η*≈0.787 as derived from the best fit of numerical simulations. Buckling stresses of the order of the gigapascal emerge. The small difference of this factor from the theoretical unit value, which corresponds to the case of simple tubular section^41^,^42^, can be imputed to the higher complexity of the buckling deformation mechanism at the central joint, which involves also multiple layers constituting the tube wall. Figure 5b shows the dimensionless moment versus rotation curves for all the analysed cases compared with the analytical prediction obtained inserting *M*_bh_=2*σ*_bh_*J*/*d* into equation 1, with *γ*=0.44. The collapse of all the curves into a single master curve confirms the validity of this last scaling-shape law. It could also be used to include statistical variation in the tetrapods geometry for the modelling of realistic networks.

### Compressive and tensile behaviour of tetrapods

**Figure 6:**
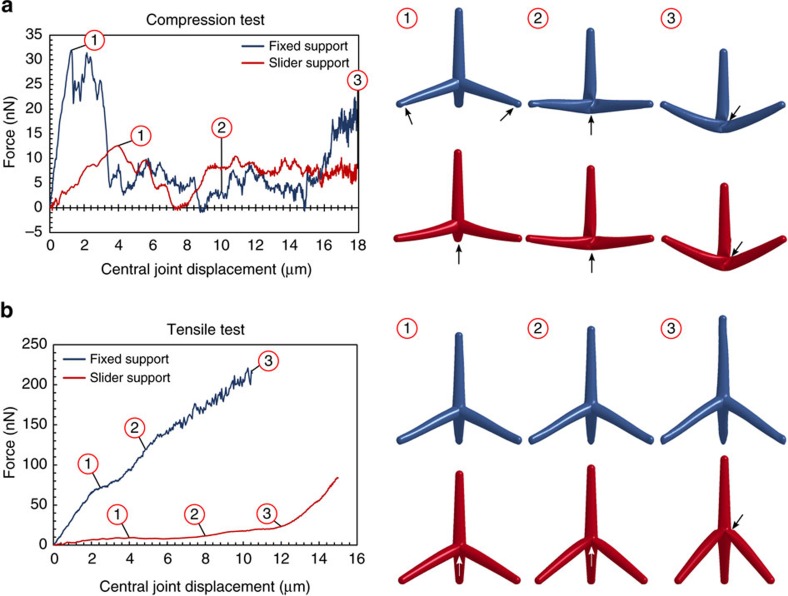
Force–displacement curves of a single tetrapod under compression or tension and fixed or sliding boundary conditions as computed by FEM simulations. The boundary configuration in the FEM images is identified by the tetrapod colour according to the graph legend. The locations of the buckling-hinge formations are highlighted with the arrows. (**a**) Compression tests showing a typical snap-through-like global instability under displacement control. The reactive moments at the clamps yield there to the formation of buckling-hinges which disappear for large displacement leading to the formation of a central hinge . The sliding boundary conditions led the formation of the hinge only at the central joint where the maximum moment takes place. (**b**) Tension test showing how the fixed boundary conditions do not allow the formation of a buckling hinge thus, the tetrapod behaviour is governed by arm bending. In the sliding boundary conditions case, stiffening after displacement level is due to the base arms alignment along the loading direction after the formation of the central hinge. See Supplementary Movies 6–9 of the 4 tests.

With FEM simulations we performed further mechanical characterizations on the same tetrapod geometry under pure compression or tension, with fixed or sliding boundary conditions. We subjected the central joint of the tetrapod to an imposed displacement orthogonal to the substrate. The results are reported in Fig. 6. Figure 6a depicts the compression behaviour: the buckling-hinge local instability leads to a global snap-through instability. For sliding boundary conditions the buckling-hinge appears at the central joint while for fixed boundary conditions early buckling-hinges appear on each arm near the clamps (deformation level ), being there also a bending moment. At larger joint displacement the central buckling-hinge occurs (level ) while the arm hinges disappear. After the snap-through the three base arms are under tension and a further increase in the force is observed (level ). Regarding the tensile behaviour depicted in Fig. 6b, the fixed boundary conditions are able to prevent buckling and the tetrapod behaviour is governed by the elastic bending of the arms attached to the substrate, thus resulting in much higher overall stiffness and bearing capacity with respect to the sliding boundary conditions. In the latter, the formation of the buckling-hinge at the central joint is observed, representing an example of buckling in tension^43^; at very large displacements the tetrapod starts to stiffen, being governed by the arms axial rather than bending stiffness. The slope of the force–displacement curve is nearly the same in both tension and compression as expected, and depicted in the Supplementary Fig. 3. The four in-silico tests—related Supplementary Videos 6–9 are provided—, which could be considered as limiting cases of real scenarios where mixed boundary conditions are expected (compliant clamps), are all in agreement with the buckling-hinge model prediction, as demonstrated in the Supplementary Fig. 4, confirming the generality of the proposed approach.

## Discussion

Complex shaped hollow nano- and micro-structures, for instance the here considered tetrapods, enable the tunable fabrication of advanced 3D highly porous materials with unique mechanical specific properties. The non-linear constitutive law of these modular networks is mainly dictated by the mechanical behaviour of the individual network building blocks, which themselves strongly depend upon their morphology. In particular, the mechanics of single hollow AG tetrapods with hollow arms, that in the present work have been successfully synthesized from sacrificial ZnO tetrapods in a single-step CVD conversion process, is governed by the buckling-hinge formation at the central joint or along its arms, rather than by the elastic deformation of the arms, as dictated by its thin walls. This mechanism, which clearly emerges from experiments and simulations, is reversible and allows high overall deformation without damage under extreme and cyclic loads, as confirmed and visible by experiments. The developed analytical model, which describes the mechanical behaviour of the tetrapod buckling-hinges with three parameters (the arm rotation at buckling onset *α*_bh,_ the hinge elastic rotational stiffness *D* and the buckling-hinge parameter *γ*), represents the essential basis for understanding the mechanical behaviour of AG networks as a whole. The nonlinear softening of tetrapods suggests that the relative network under compression may experience an analogous behaviour before its stiffening due to material densification. Indeed, this is in agreement with the experimentally observed behaviour for networks, which shows a change in the sign of the stress–strain curve second derivative (non-linear softening followed by stiffening)^27^. We believe that our findings on the dominant deformation mechanisms of individual AG tetrapods can lead to a more profound understanding of the mechanical behaviour of the 3D interconnected t-AG. Moreover, due to the proved generality of the buckling-hinge model, not restricted to the specific geometry, loading and boundary conditions, size-scale, and shape of the tetrapod, our work is expected to be useful in the design and optimization of aerogels and foams^44^ in different fields, from materials science to scaffold medical engineering.

## Methods

### Production of aerographite

The carbon-based AG tetrapods with hollow microtubular arms were synthesized by direct conversion of sacrificial zinc oxide nano- and micro-tetrapods^27^ at 760 °C by a CVD process described in a previous work^15^. In the CVD process, networks of μm-sized tetrapodal ZnO (92% porosity)^21^ were converted into AG networks (*V*∼1 cm^3^) equipped with nm-thick, closed multilayer graphene shells. The morphology of the AG network was studied using an Ultra Plus Zeiss SEM (7 kV). Several carbon tetrapods were carefully selected from a single AG 3D network sample for *in situ* bending investigations in the SEM. The bending experiments were done using *in situ* methods in a Hitachi SEM S-4800. For manipulation of the free-standing tetrapods a customized piezo-driven SmarAct 13D nanomanipulation system^45^,^46^,^47^ was used. To measure their mechanical properties, soft AFM cantilevers BL-RC-150VB from Olympus (spring constant *k=*2.9–50 pN nm^−1^) were utilized. Two different types of bending experiments were performed which are described below.

### Single tetrapod bending experiments

In the first *in situ* experiment, tetrapods were dropped on a Si/SiO_2_ wafer after scratching them from a bulk AG sample. For mechanical measurements, only the tetrapods which were strongly adhering by vdW forces with the wafer substrate were chosen, that is, those attached with three arms to the substrate and thus did not change position during the bending experiments. When the proper geometrical alignment was achieved, the tip of the cantilever was moved towards the free arm of the tetrapod and the bending of both tetrapod and cantilever was observed. The whole motion was captured in a video (see Supplementary Movies 1 and 2), which was analysed frame-by-frame, to quantify the deflections of both, tetrapod and cantilever, respectively (calculation of the torque–deflection-curves from the *in situ* video data is described in detail in the Supplementary Note 1).

### Isolated tetrapod arm bending experiments

In the second *in situ* experiment, tetrapods were glued, using a micromanipulator, with a conductive epoxy (CW2400) to an electrochemically etched gold tip, thus forming a small network of AG at the apex of the tip. Afterwards a part of a single arm of a tetrapod was picked up from the network with another gold tip, which actually was possible due to the strong vdW interactions between the gold tip and the AG tube. Then the single free tetrapod arm was brought into contact with a third Au tip and the buckling experiment was performed. In these measurements, the tetrapod was buckled against two approaching gold tips (Fig. 4a–d) and the mechanical deformation was observed and evaluated by analysing the videos recorded during the experiment (see Supplementary Movie 4 for the *in situ* experiment and Supplementary Movie 5 for its simulation).

### Buckling-hinge model

The model was build making the analogy with the plastic hinge formation in bent beams. In the most general expression the behaviour of a section forming a local buckling-hinge can be expressed substituting plastic characteristic thresholds with the buckling counterparts:


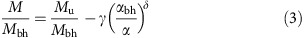


where *α*_bh_ is the joint (hinge) rotation at the buckling onset, *M*_u_ is the ultimate asymptotic moment that the hinge is able to carry, *δ*>0 and 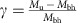 since *M* (*α*=*α*_bh_) ≡ *M*_bh_ for continuity. Thus we can compute the evolution of the joint stiffness in the nonlinear regime as derivative of the *M*–*α* relationship. It follows:


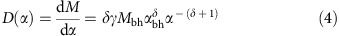


Note that 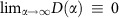 and that for guaranteeing continuity of the curve slope *D*(*α*) at the buckling onset (*α*=*α*_bh_) it must hold *δγ*=1. We come then to the final formulation of the nonlinear buckling-hinge law of equation (1). For modelling the evolution of the shape of the arm cross-section which is assumed to be elliptical with major and minor semi-axes *a* and *b*, respectively, the two conditions of section perimeter conservation and the relation of the buckled cross section inertia to the current value of the joint rotational stiffness *D* are expressed by the following equations, that must hold for each arm bending angle:


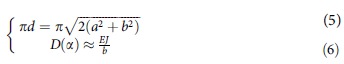


where *d* is the diameter of the tube before buckling in the section where the hinge forms. The system of the two previous equations can be solved numerically state by state providing the evolution of the cross sectional shape after buckling (Fig. 4e). The result at buckling onset is analogous to the one that can be derived by different method presented elsewhere^35^.

### Finite element simulations

FEM models of the tetrapods (results reported in Figs 2, 4 and 6) were built associating the arms extremities and the central joint of the tetrapods to the vertexes and centroid of a regular tetrahedron, respectively. The tube walls were modelled with thin shell elements with selective-reduced integration^48^, while the spurious modes effects were properly controlled. For the bending experimental set-up three arms were fully clamped at the end accounting for the adhesion to the substrate, while both fixed and sliding boundary conditions were considered for both compression and tensile tests. The constraint is applied to a set of nodes rather than a single point, to avoid undesired stress localization and to properly account for moments at the clamped restrains. The arm deflection simulating the AFM load is obtained imposing the displacement at the end of the arm, in order also to maximize and quantify the contribution of the arm bending stiffness with respect to the one of the rotational stiffness of the tetrapod central joint. In compression and tension tests the displacement is imposed at the intersection node of the arms axes (tetrahedron centroid). The total applied force, and the bending moment at the joint are computed from resultant at the restrained node with the substrate. Self-contact is implemented to avoid walls interpenetration at the buckling/folding sites due to large displacements and properly evaluate the post-buckling contribution. The model for the single arm buckling (second type of *in situ* experiment) follows the same procedure, with the arm modelled as a clamped cantilever at one of the ends (see Supplementary Movie 5) and subjected to a transversal imposed displacement at the tip simulating the action of the gold manipulator. The critical buckling point (*M*_bh_, *α*_bh_) is determined looking at the evolution of the tetrapod deformation energy *U*, in particular it corresponds to the drop in the local derivative of the *U*–*α* curve^42^.

### Data availability

The data that support the findings of this study are available from the corresponding authors upon request.

## Additional information

**How to cite this article:** Meija, R. *et al*. Nanomechanics of individual aerographite tetrapods. *Nat. Commun.* **8**, 14982 doi: 10.1038/ncomms14982 (2017).

**Publisher's note:** Springer Nature remains neutral with regard to jurisdictional claims in published maps and institutional affiliations.

## Supplementary Material

Supplementary Information Supplementary Figures, Supplementary Notes and Supplementary References

Supplementary Movie 1 First type of in situ experiment showing the formation of a buckling-hinge at the central joint of a tetrapod with three arms fixed at a substrate and one being bent by an AFM cantilever. This video was used as basis for the curves shown in figures 2 and 3 in the main text.

Supplementary Movie 2 Another example for the first type of in situ experiment executed on a second tetrapod obtained from the same sample.

Supplementary Movie 3 FEM simulation corresponding to the experiment shown in Supplementary Video 1

Supplementary Movie 4 Second type of in situ experiment showing the formation of a buckling-hinge in a single AG tube clamped between two gold tips.

Supplementary Movie 5 FEM simulation corresponding to the experiment shown in Supplementary Video 4

Supplementary Movie 6 FEM simulation of tetrapod test in compression with fixed boundary conditions.

Supplementary Movie 7 FEM simulation of tetrapod test in compression with sliding boundary conditions.

Supplementary Movie 8 FEM simulation of tetrapod test in tension with fixed boundary conditions.

Supplementary Movie 9 FEM simulation of tetrapod test in tension with sliding boundary conditions.
