# Supplementary material for: Nanomechanics of individual aerographite tetrapods
Source: Nat Commun. 2017 Apr 12;8:14982. doi: 10.1038/ncomms14982 (PMC5394344; doi:10.1038/ncomms14982)
Supplement: Supplementary Information — Supplementary Figures, Supplementary Notes and Supplementary References [file ncomms14982-s1.pdf]

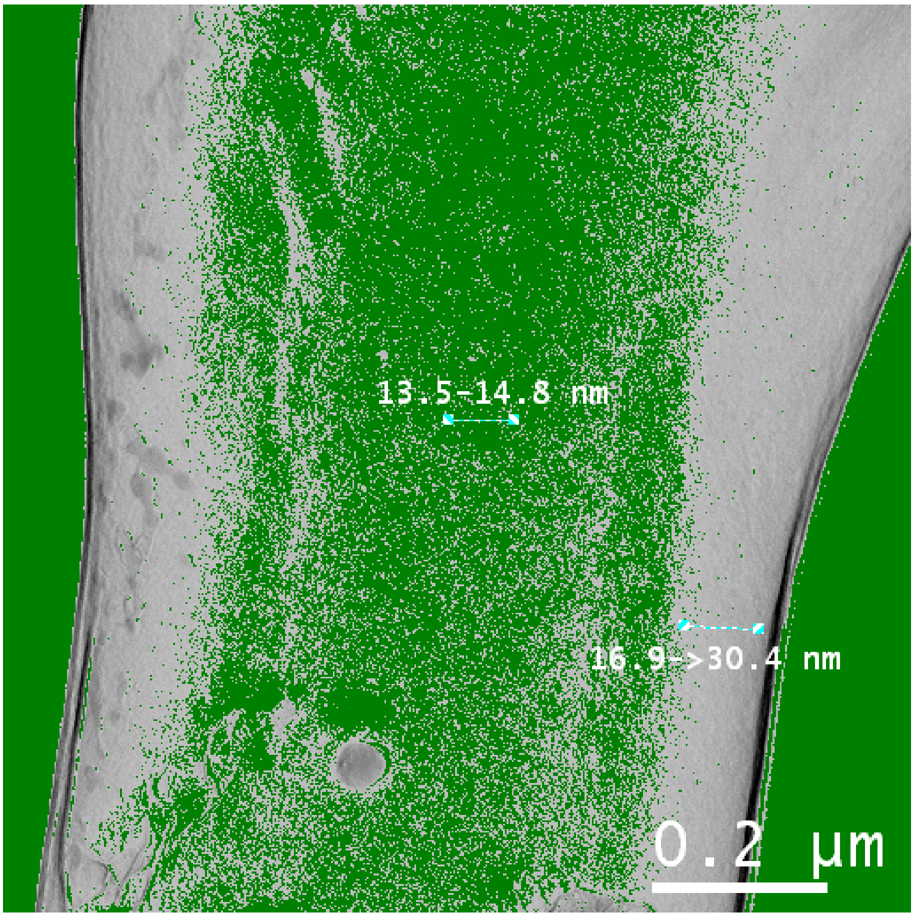

Supplementary Figure 1 | By analyzing the energy filtered TEM (EFTEM) images according to the log-ratio technique,[1] representative mappings of the wall thicknesses of tubular AG ligaments were created. The example shown here exhibits a wall thickness of about 15 nm which is typical for the AG samples with densities in the range of 1-5 mg cm^{-3} which were used for this study.

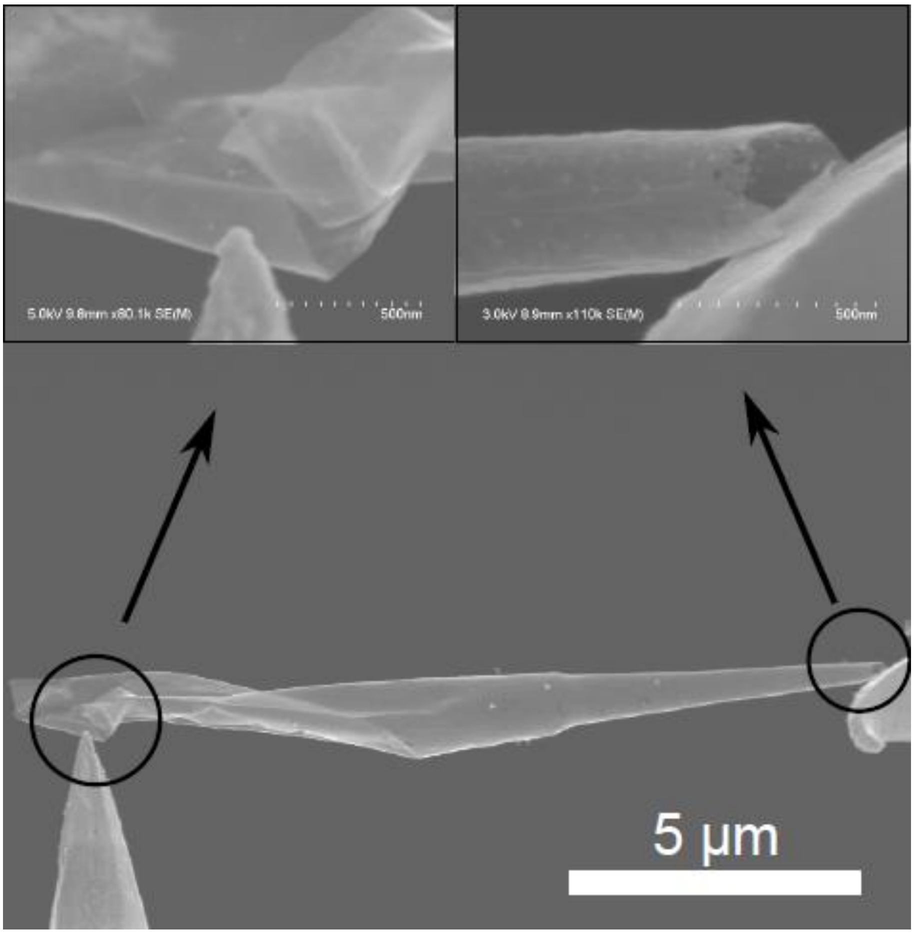

Supplementary Figure 2 | SEM micrograph of an individual aerographite tube isolated from the network and contacted with two gold tips. The insets show a magnified view of the contact points between gold tip and tube for the electrical measurements.

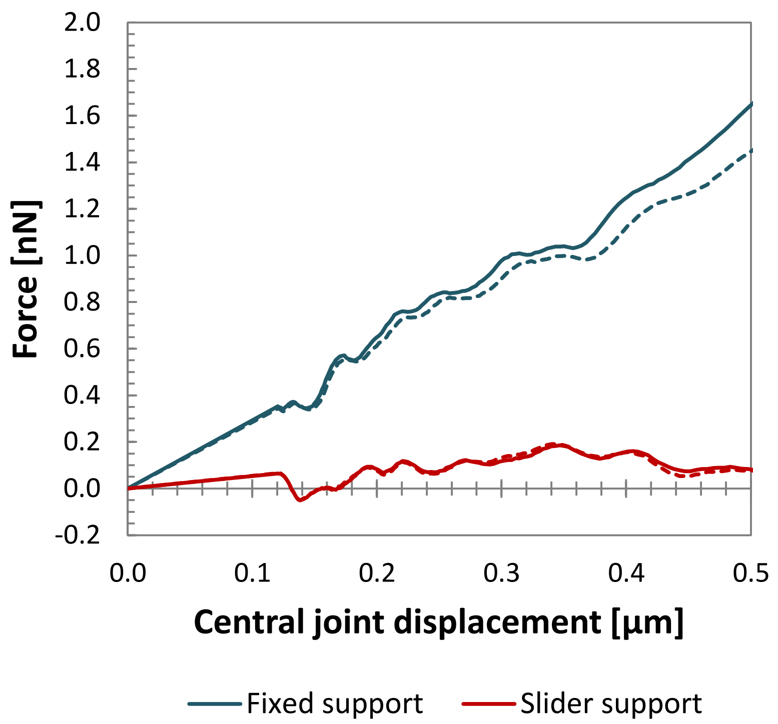

Supplementary Figure 3 | Magnifications at small displacements of the force-displacement curves reported in Figure 6 in the main text of tetrapods under compression or tension and fixed or sliding boundary conditions ($\varepsilon = \Delta h/h_0 < 1.3$ % where $h_0 = 37.5$ μm is the initial total height of the tested tetrapod). Compression and tensile behaviour (dashed and continuous lines respectively) are compared for the two different boundary conditions, confirming that prior to the nonlinear regime (buckling) the tetrapod stiffness is the same in tension and compression.

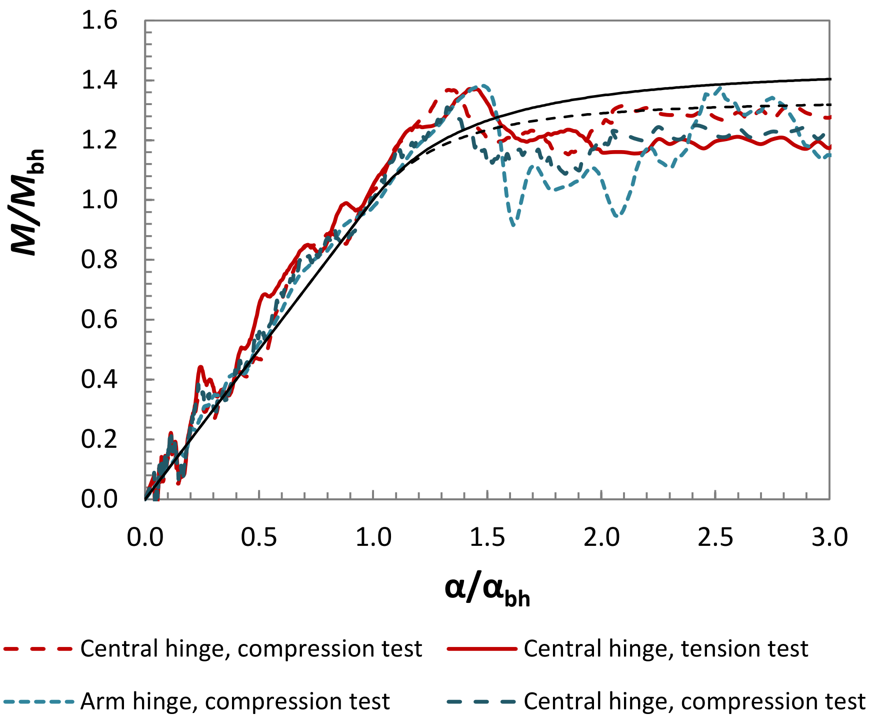

Supplementary Figure 4 | Dimensionless moment-rotation curves for the buckling hinges formation in the tetrapod. Buckling hinges appear in the central joint and/or close to the arms near the clamps. Compression or tension tests and sliding or fixed boundary conditions are considered. Red curves correspond to tension or compression tests with sliding boundary conditions; the blue lines refer to the two hinges appearing in the compression test with fixed boundary conditions (see Figure 6a in the main text and Supplementary Video 6). No hinge formation is observed for tensile test with fixed boundary conditions (Figure 6b in the main text). The constitutive behaviour of all the buckling hinges is well described by the model prediction. The black lines represent the fit of the buckling hinge model (Equation 1 in the main text) to the AFM tetrapod bending experiments (continuous line is related to the experiments reported in Figure 3 in the main text, γ=0.44, whereas dashed line is related to the experiments reported in Figure 4 in the main text, γ=0.33). Thus, these results -related to different loading and boundary conditions- confirm the generality of the proposed model without invoking any best fitting parameters.

Supplementary Note 1: Calculation of the moment-rotation curves from videos of *in situ* experiments

The tip of the atomic force microscopy (AFM) cantilever was moved towards the free arm of the tetrapod and the bending of both tetrapod and cantilever was observed. The whole motion was captured in videos. Supplementary Video 1 shows the bending of the tetrapod that corresponds to the FEM model case shown in figures 2 and 3 in the main text and in the Supplementary Video 3, while Supplementary Video 2 shows the joint deformation of a further analysed tetrapod from the same sample. For the computation of the moment-rotation curve it was necessary to determine the real arm length and deflection of the cantilever because in the recorded images just the projection of both is visible. Therefore the tilting angle of the tetrapod arm and of the cantilever with respect to the optical axis of the SEM was calculated. For the AFM cantilever the total length is known and the tilting angle was determined by Supplementary Equation 1 where L_{real} denotes the total length of the cantilever and L_{proj} denotes the projected length of the cantilever which is visible in the SEM micrograph. It follows:

$$\alpha = arcsin\left(\frac{L_{\mathrm{real}}}{L_{\mathrm{proj}}}\right) \tag{1}$$

The same formula holds for the tilting angle of the tetrapod arm but in this case it was necessary to determine the total length of the tetrapod arm first. In order to determine this length, a series of SEM micrographs with different tilting angles of the SEM stage was recorded. By plotting the projected length of the tetrapod arm against the tilting angle of the stage and the application of a sinusoidal curve fit, the total arm length was determined. Subsequently, the angle of the AFM cantilever relative to the tetrapod arm and the torque which acted on the tetrapod arm was calculated. The torque moment is the vector product of the lever arm **r** and the force vector **F**, thus:

$$M = |\mathbf{r}||\mathbf{F}|sin\theta \tag{2}$$

In the calculations, **r** was defined as the connection line between the basis of the arm at the tetrapod junction and the projection on the arm axis of the contact point with the AFM cantilever tip. This means, the torque is supposed to attack at the point where the tetrapod arm

is attached to the junction and not at the center of the tetrapod. The reason for this data evaluation was the observation, that the tetrapod junction gets distorted during the bending.

Supplementary references

1. Malis, T., Cheng, S.C. & Egerton, R.F. The EELS log-ratio technique for specimen-thickness measurement in the TEM. *J. Electron Microsc. Tech.* **8**, 193–200 (1988).
